# Supplementary material for: Association between Depression, Anxiety Symptoms and Gut Microbiota in Chinese Elderly with Functional Constipation
Source: Nutrients. 2022 Nov 25;14(23):5013. doi: 10.3390/nu14235013 (PMC9740187; doi:10.3390/nu14235013)
Supplement: Supplementary file 1 [file nutrients-14-05013-s001.zip › nutrients-1989860-supplementary.pdf]

Association between depression, anxiety symptoms and gut microbiota in Chinese elderly with functional constipation.

Supplementary Materials:

Table S1: Correlation of depression and anxiety with other influencing factors. (n = 198, %)

	Total	PHQ-9 Mild 61 (30.8)	χ^2	p	GAD-7 Mild 43 (21.7)	χ^2	p
BMI (kg/m²)			14.275	**0.001**		9.252	**0.010**
< 20	19	13 (68.4)			9 (47.4)		
20.0-26.9	140	39 (27.9)			29 (20.7)		
> 26.9	39	9 (23.1)			5 (12.8)		
PSQI			35.274	**< 0.001**		6.901	**0.009**
Good	146	28 (19.2)			25 (17.1)		
Poor	52	33 (63.5)			18 (34.6)		
High dietary fiber intake			12.973	**0.002**		2.417	0.299
Low	68	30 (44.1)			16 (23.5)		
Moderate	65	21 (32.3)			17 (26.2)		
High	65	10 (15.4)			10 (15.4)		
IPAQ			8.770	**0.012**		5.068	0.079
Low	48	23 (47.9)			16 (33.3)		
Moderate	120	31 (25.8)			22 (18.3)		
High	30	7 (23.3)			5 (16.7)		

BMI: Body mass index. The Pittsburgh Sleep Quality Index, PSQI. The International Physical Activity Questionnaire, IPAQ. Compared using chi-square test. Bold values indicate statistically significant values, $p < 0.05$.
